# Gender Differences in Cardiovascular Risks of Obese Adolescents in the Bronx

**DOI:** 10.4274/jcrpe.v2i2.67

**Published:** 2010-05-03

**Authors:** Ping Zhou, Ronak S. Chaudhari, Zoltan Antal

**Affiliations:** 1 Pediatric Endocrinology, Children’s Hospital at Montefiore, Albert Einstein College of Medicine, Bronx, NY, United States; 2 Department of Internal Medicine, North General Hospital, Mount Sinai School of Medicine, New York, NY, United States; 3 Division of Pediatric Endocrinology, Cornell University, Weill Medical College, New York, NY, United States; +90 718 920−4664+90 718 405−5609pzhou@montefiore.orgMD, Assistant Professor, Division of Pediatric Endocrinology, Albert Einstein College of Medicine, 3450 Wayne Ave., Bronx, NY 10467, USA

**Keywords:** obesity, cardiovascular disease, gender, Pediatrics, adolescents

## Abstract

**Objective**: The associations between the degree of obesity and cardiovascular disease (CVD) risks and the impact of gender differences with regard to these risk factors are not well understood. The aim of our study was to examine the gender−specific differences in CVD risk factors in inner−city minority youths.

**Methods**: A total of 269 adolescents (109 males and 160 females) were included in this retrospective study. Data on multiple metabolic variables were collected. Evaluation of abnormalities in these parameters was based on standard criteria. Pearson correlations were calculated to examine the relationship between body mass index (BMI) z−score and obesity−related parameters. Chi−square and Fisher’s tests were used to compare the frequencies of single or multiple cardiovascular risks in the two gender groups.

**Results**: In the male group, BMI z−scores showed significant positive correlations with insulin resistance and diastolic blood pressure. In the female group, BMI z−scores showed significant positive correlations with insulin resistance and systolic blood pressure. In both genders, the prevalence of CVD risks was high, but a similar risk pattern was established in both sexes, with dyslipidemia being the highest, hypertension the second, and impaired glucose tolerance (IGT) being the least outstanding. The most important finding in this study was that the male group had a significantly higher prevalence of systolic hypertension.

**Conclusion**: Male inner−city minority adolescents show a high prevalence of CVD risks associated with obesity. Appropriate risk stratification is critical to developing and implementing both therapeutic and preventive interventions.

**Conflict of interest:**None declared.

## INTRODUCTION

The Bronx is the poorest urban county in the country −50% of Bronx households include young children living below the poverty level. 95% are Black and Latino, including African−Americans, Caribbean−Americans, and new immigrants from Africa. Residents there suffer from high rates of diabetes, obesity, and depression (1).

According to the most recent United States (US) epidemiologic data (2), the prevalence of obesity among adolescents is 15.5%. The Bronx has a much higher prevalence rate, which reaches 25%. Excessive body weight predisposes children to many cardiovascular disease (CVD) risks; and can lead to hypertension, dyslipidemia, impaired glucose metabolism, and insulin resistance in their adulthood. However, not all obese children share similar risks for developing these conditions. Factors such as race, gender, economic condition, life style as well as the duration of obesity and its degree may also play an important role for developing CVD. Although the prevalence of obesity has been increasing in both boys and girls, few studies have examined the role of gender in determining obesity−related CVD risks.

The aim of our study was to examine the gender−specific differences in CVD risk factors in obese adolescents in a high−risk area of an inner city, the Bronx.

## METHODS

This is a cross−sectional retrospective study approved by our Institutional Review Board. Inclusion criteria were obesity, age between 13 and 19 years, and a Tanner stage development of at least stage III. Exclusion criteria included underlying diseases that could cause hypertension, type 2 diabetes mellitus or conditions that were being treated with medications.

Our data were collected from records of pediatric endocrine outpatient clinic visits. The standard techniques were routinely used in the clinic. Height was measured by a stadiometer (Holtain Ltd., Crymych, and Wales) and weight by a standing scale (Scale−Tronix, Wheaton, Ill.). Arterial blood pressure was measured with a cuff of appropriate size in sitting position. Body mass index (BMI) was calculated using the following equation: BMI=weight (kg)/height^2^ (m^2^). This value then was converted to a percentile and z−score for each individual patient using standard age and sex specific charts (Department of Health and Human Services 2007). Children with a BMI percentile value of ≥95% were considered obese.

All subjects underwent a thorough clinical screening, which included history taking and physical examination. A comprehensive laboratory assessment, which included measurement of thyroid hormone, electrolytes, liver, and renal function tests, was also performed. In most subjects this assessment also included a lipid profile in the fasting state and a 2−hour oral glucose tolerance test (OGTT). All biochemical tests were performed in the Montefiore Medical Center clinical laboratory.

After screening, a total of 269 adolescents (160 females and 109 males), referred to endocrine clinics for evaluation of obesity, were included in this study. Among these children, 109 (41%) were African−American, 143 (53%) were Hispanic, and 17(6 %) were of mixed genetic background. Data on these patients, expressed as means±standard deviations, are presented in Table 1.

The parameters were assumed to be abnormal if the following criteria were met: Dyslipidemia was defined as having a cholesterol (Chol) level of >=200 mg/dL, an HDL of <=35 mg/dL, an LDL of >=130 mg/dL, or a triglyceride level of >=150 mg/dL (3). Abnormal glucose level was defined as a fasting plasma glucose level of 100−125 mg/dL and a two−hour plasma glucose level of 140−200 mg/dL (patients with overt type 2 diabetes were excluded from this study) (4). Insulin resistance (IR) was defined by the Quantitative Insulin Sensitivity Check Index (QUICKI), which is calculated as QUICKI=1/ [log (I0*G0)], and values of less than 0.34 indicate presence of IR (5). Hypertension was defined as either systolic or diastolic blood pressure (BP) being equal to or greater than the 95th percentile adjusted by age, gender, and height (6).

**Data Analysis**

The data were analyzed using a SAS statistical package (SAS Institute, Cary, NC). Pearson correlations were calculated to examine the relationships between BMI z−score and measurements including BP, glucose levels, QUICKI and lipids. Chi−square and Fisher’s tests were used to compare the frequency of single or multiple cardiovascular risks in both genders. For all tests, statistical significance was defined by p values less than 0.05.

**Table 1:**
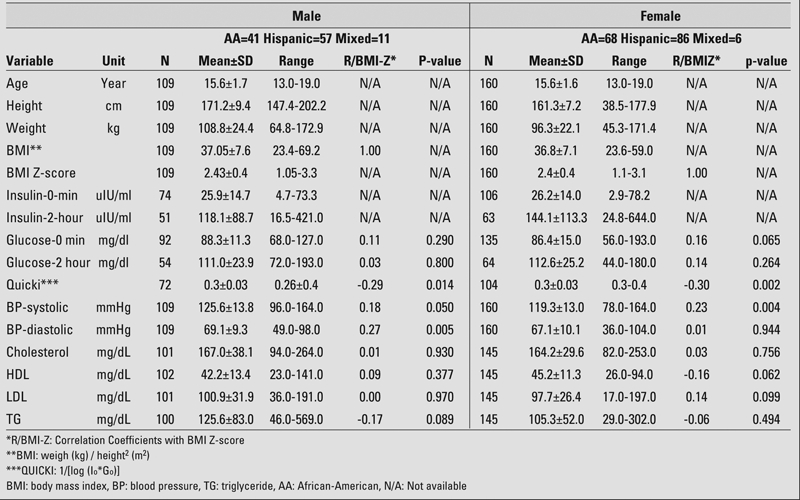
Patient data

## RESULTS

In the male group, BMI z−score showed a significant negative correlation with QUICKI (r=−0.28 and p=0.014). Both diastolic and systolic blood pressures were positively correlated to BMI z−score, the correlations being significant for diastolic blood pressure (BP−dias) (r=0.265 and p=0.005) and fairly significant for systolic blood pressure (BP−sys) (r=0.18 and p=0.05).

In the female group, BMI z−score showed a significant negative correlation with QUICKI (r=−0.29 and p=0.002) and a positive correlation with BP−sys (r=0.225 and p=0.004). BMI z−score was also positively correlated with BP−dias, but did not reach statistical significance.

In both groups, BMI z−score correlated positively with both fasting plasma glucose and two−hour plasma glucose levels during OGTT, but neither correlation reached a significant level. BMI z−score had no meaningful associations with any parameters of lipid profile. Patient data are presented in Table 1.

**Figure 1:**
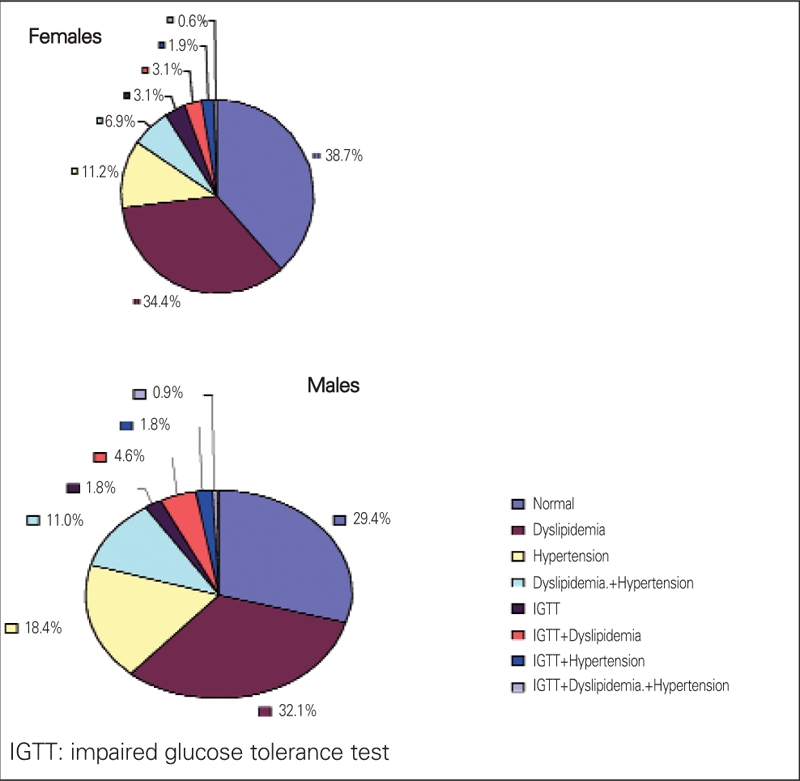
Frequency of combined CVD risks

In both gender groups, the prevalence of CVD risks is high. There is a similar pattern for each gender, with dyslipidemia standing out as the highest risk factor (48.6% for males and 45.0% for females). Hypertension ranks second (32.1% and 20.6% for males and females, respectively, p= 0.03), and impaired glucose tolerance (IGT) ranks as the lowest (9.2% for males and 8.8% for females). The co−morbidity of combined hypertension and dyslipidemia is 11.1% for males and 6.9% for females. The co−morbidity of combined hypertension and IGT is 1.8% for males and 1.9% for females. The co−morbidity of combined IGT and dyslipidemia is 4.6% for males and 3.1% for females. The co−morbidity of hypertension, dyslipidemia, plus IGT is approximately 0.9% for males and 0.6% for females. Overall, only 38.8% females and 29.4% males had no abnormalities. The male group had a significantly higher prevalence of systolic hypertension (29.6% vs. 18.8%, p=0.0057). Detailed data are shown in Table 2 and Figure 1 and 2.

**2:**
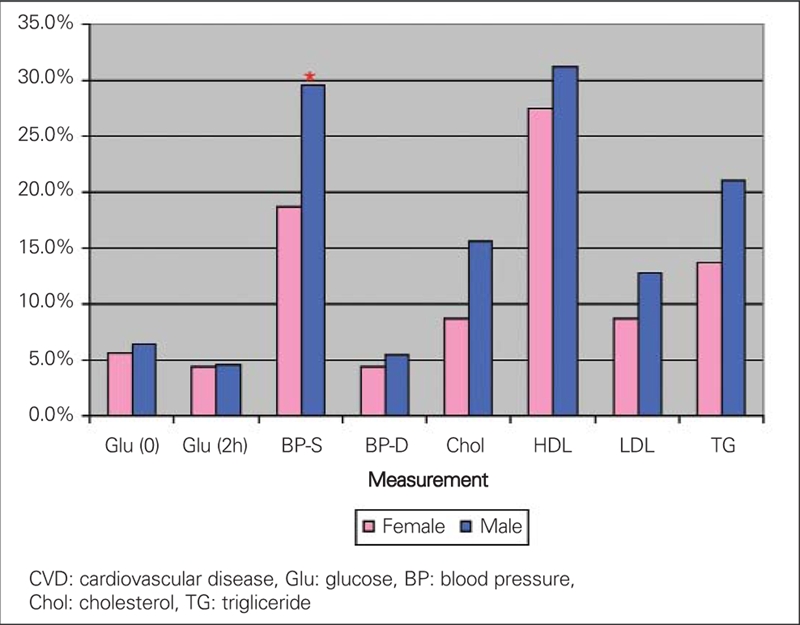
Frequency of single CVD risks

**Table 1:**
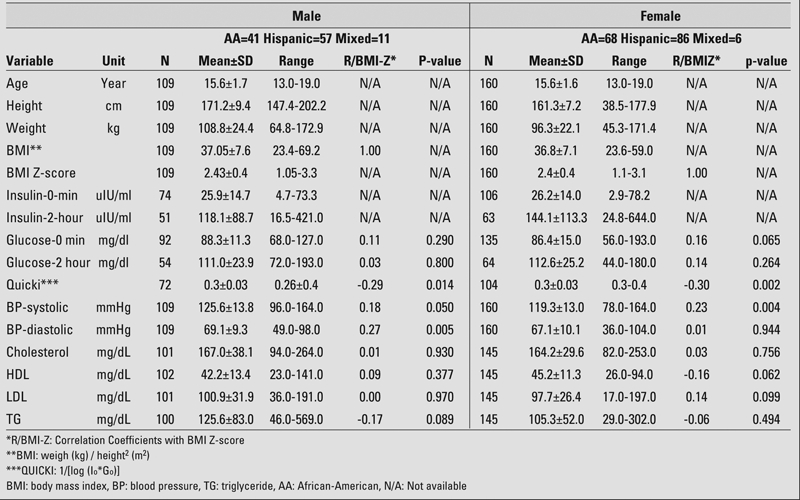
Patient data

**Table 2:**
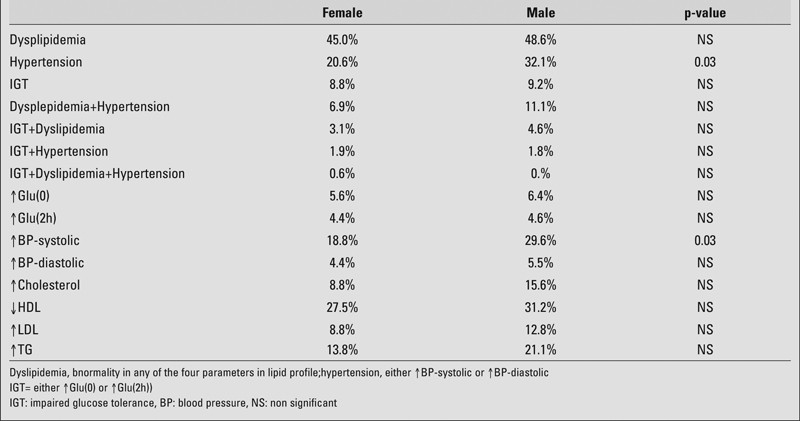
Comparison of CVD risk prevalence between genders

## DISCUSSION

In this study, we examined CVD risks of obese adolescents residing in one of the poorest inner cities of the US. The subjects consisted mainly of African−Americans and Caribbean−Hispanics. Using BMI z−score, we studied the varying degrees of obesity associated with various risk factors for CVD. Our results showed an important relationship between BMI z−score and IR, namely, the higher the BMI z−score, the worse IR. Previous studies in adults demonstrated IR as the main cause for developing CVD (7, 8). BMI z−scores have a significant impact on BP−sys in females and a greater impact on BP−dias in males. Although gender differences in childhood obesity have not been fully comprehended, previous studies indicate that the causes and consequences of obesity might differ between genders (9).

The prevalence of dyslipidemia and hypertension observed in this present study are much higher than those reported by previous studies (4, 5, 6). One interesting finding is that dyslipidemia does not seem to be linked with the degree of obesity. However, further studies on the etiology of dyslipidemia are needed to explain this finding. Also, the impaired OGTT rate in this study is lower than the rates reported in several previous studies (10, 11). Given that this is a retrospective study and not all subjects took the OGTT, this finding may have resulted from the smallness of the sample size for this test. Most interestingly, we found that males carry higher risks of systolic hypertension.

## CONCLUSION

Male subjects among inner−city minority youths carry higher CVD risks associated with obesity, specifically with regard to systolic hypertension. An awareness of this gender difference is critical in developing and implementing both therapeutic and preventive interventions in this population.

**Acknowledgments**

We thank Jennifer M Camacho and Judith Green, both of which are medical students at Albert Einstein College of Medicine, for collecting partial data in this study. We also thank Dr. Morri Markowitz, MD, Professor of Pediatrics, Division of Pediatric Endocrinology, and Dr. Peter F Belamarich, Professor of Pediatrics, Division of Pediatric Gastroenterology, at Albert Einstein College of Medicine, for many helpful suggestions and reviewing this manuscript. Finally, we want to show our gratitude for the help with statistical analysis by Ziyong Cai, Ph.D., Director of Specialized Analytics at Decision Management of Citigroup.
